# Quality of life and its determinants in women with delayed conception in low-mid socioeconomic neighbourhoods of Northern India: a cross-sectional study

**DOI:** 10.1136/bmjph-2024-001740

**Published:** 2025-04-20

**Authors:** Barsha Gadapani Pathak, Gitau Mburu, Ndema Habib, Rita Kabra, James Kiarie, Ranadip Chowdhury, Neeta Dhabhai, Sarmila Mazumder

**Affiliations:** 1 Society for Applied Studies, New Delhi, New Delhi, India; 2 Centre for International Health, Faculty of Medicine, University of Bergen, Bergen, Norway; 3 UNDP-UNFPA-UNICEF-WHO-World Bank Special Program of Research, Development and Research Training in Human Reproduction (HRP), Department of Sexual and Reproductive Health and Research, World Health Organization, Geneva, Switzerland

**Keywords:** Public Health, Preventive Medicine, Social Medicine, Epidemiology

## Abstract

**Background:**

The inability to conceive or delay in conception has negative and multidimensional effects on health and well-being, daily functioning and societal interactions. This study assesses the impact of delayed conception on quality of life (QoL) among Indian women with delayed conception and evaluates the Fertility Quality of Life (FertiQoL) questionnaire’s reliability and construct validity in this population.

**Methodology:**

A cross-sectional study of 1530 women aged 18–30, who had not conceived over a period of 18 months, was conducted in low-to-mid-socioeconomic neighbourhoods in Delhi, India. The 24-item core module of the FertiQoL questionnaire was used to assess participants’ QoL. Researchers analysed data to identify factors associated with QoL scores and evaluated FertiQoL’s internal consistency and validity. Data were collected between July 2020 and August 2021.

**Result:**

The average FertiQoL score was 31.71 out of 100, indicating a moderately low QoL. Among the subscales, emotional (mean: 29.0) and mind-body domains (mean: 25.4) scored the lowest, while the relational domain scored the highest (mean: 50.7). Factors negatively associated with QoL included a longer duration of delayed conception (β=−0.3, 95% CI: −0.5 to −0.1), husbands fathering children with other partners (β=−1.1, 95% CI: −1.7 to −0.38), domestic violence—emotional (β=−3.5, 95% CI: −4.5 to −2.4), verbal (β=−3.2, 95% CI: −4.7 to −1.7), physical abuse by family (β=−3.6, 95% CI: −5.5 to −1.6), lack of financial support (β=−3.2, 95% CI: −5.2 to −1.2), social pressures (β=−1.6, 95% CI: −2.3 to −0.9) and physical health issues (β=−0.9, 95% CI: −1.7 to −0.2). The FertiQoL tool demonstrated acceptable reliability (Cronbach’s alpha=0.64) and good construct validity (confirmatory factor analysis), confirming its utility in this population.

**Conclusion:**

Indian women experiencing delayed conception have reduced QoL, particularly in emotional and mind-body domains, due to various psychosocial and socioeconomic challenges. The FertiQoL questionnaire proved to be a reliable and valid tool for assessing QoL in this context. Psychosocial interventions addressing emotional, social and economic stressors are urgently needed to improve their well-being. Future research should include men to better understand the holistic challenges faced by couples struggling to conceive.

**WHAT IS ALREADY KNOWN ON THIS TOPIC**

- The inability to conceive or delayed conception negatively impacts health, well-being, daily functioning and social interactions. The Fertility Quality of Life (FertiQoL) questionnaire is a globally validated tool for assessing the quality of life (QoL) in individuals with infertility/delayed conception, but its application in Indian communities has been limited.

**WHAT THIS STUDY ADDS**

- This study reveals that women experiencing delayed conception in low-mid socioeconomic neighbourhoods of Northern India have a moderately lower QoL, particularly in emotional and physical domains. Factors such as the duration of attempting to conceive, husband fathering a child with another partner, domestic violence and social pressures significantly affect QoL. The FertiQoL questionnaire demonstrated acceptable reliability for this population.

**HOW THIS STUDY MIGHT AFFECT RESEARCH, PRACTICE OR POLICY**

- The findings highlight the need for psychosocial support to enhance the QoL for women facing delayed conception in India. Interventions should target the emotional, social and economic challenges associated with delayed conception. Healthcare providers should be aware of the negative impacts on patients’ emotions, relationships, physical well-being and social aspects and should tailor their approach to better support patients. This study also establishes the FertiQoL questionnaire’s reliability for Indian women and urges future research to include men in studies of infertility, in order to provide a comprehensive understanding of the challenges faced by couples trying to conceive.

## Introduction

 Being unable to achieve pregnancy is a complex issue that impacts many couples. Although the duration to achieve pregnancy can vary, the failure to achieve pregnancy after 12 months of regular unprotected sexual intercourse is defined as infertility.^1 2^ Globally, approximately 17.5% of couples experience infertility during their lifetime.^3^ In India, the prevalence of couple infertility reaches up to 15%.^4^,^6^

Despite procreation being an individual choice,^7^ it is frequently marred by societal pressures.^6^ In many Indian communities, childbearing is seen as a marker of a woman’s worth, and women are often held solely responsible for infertility or delayed conception, even when male factors contribute equally to the issue. Failure to achieve pregnancy can cause psychological and social difficulties including stigma, marital problems, depression and anxiety.^8 9^ In addition, the low availability and high cost of most infertility/delayed conception treatments also present challenges to people, especially in low and middle-income countries (LMICs).^10 11^ As a result, only half of the couples with infertility seek help.^12^ Of those who seek treatment, only about half conceive and one-quarter of infertility cases remain undiagnosed even after investigation.^12^,^14^ These challenges have cumulative effects, and as a result, the impact of infertility/delayed conception is wide, affecting the holistic well-being and quality of life (QoL) of individuals and couples.^15^

The WHO defines QoL as ‘an individual’s perception of their position in life in the context of the culture and value systems in which they live’.^16^ In India, where fertility is often considered central to a woman’s role within the family and society, the inability to conceive is associated with significant social stigma and emotional distress.^9^ Given that people’s social position is often dependent on their fertility status, in many contexts,^6^ understanding the links between delayed conception and QoL becomes relevant, particularly in LMICs where interventions for prevention, diagnosis and treatment of delayed conception are limited.^17 18^

Numerous studies have explored the relationship between QoL and delayed conception among both men and women and demonstrated that delayed conception has indeed a negative impact on QoL.^19^ However, the ways in which delayed conception and QoL are linked are rarely explored in India.^20^,^22^ The subtle and multidimensional effects of infertility/delayed conception on holistic health, daily functioning and societal interactions in the Indian context remain inadequately explored, and this is particularly relevant for India. This gap is especially relevant in contexts where fertility is predominantly viewed as a women’s issue, significantly impacting their QoL, and where studies have demonstrated this fact.^19^ In India, many communities place significant importance on women’s ability to reproduce,^23^ often within 1 year of marriage.^24^ Fertility is perceived as a women’s issue and where patrilocal residence,^24 25^ that is, a cultural and social practice where married couples reside with or near the husband’s family, typically in his parental home or ancestral village, amplifies familial and societal pressures. Studies also indicate that infertility/delayed conception impairs QoL more significantly among women compared with men.^19^

Given the above research gap, this study aimed to evaluate the QoL and its correlated factors among women experiencing delayed conception. By addressing this objective, this research seeks to fill a critical gap in understanding the impact of delayed conception on QoL in India, potentially increasing awareness and informing the development of culturally sensitive interventions and support programmes for infertile couples.

An additional objective of this study is to assess the psychometric properties of the Fertility Quality of Life (FertiQoL) questionnaire, including its internal consistency, reliability and validity. Earlier, self-assessment tools were employed to evaluate the QoL in individuals dealing with the inability to conceive. Nevertheless, numerous current questionnaires addressing delayed conception distress and treatment response fail to fulfil the specific requirements unique to fertility issues.^26^ Recognising the limitations of general QoL instruments, researchers developed the FertiQoL questionnaire, a multidimensional tool assessing emotional well-being, social functioning, physical health, relationship dynamics and financial concerns.^27^,^29^ While widely accepted and validated in diverse cultural contexts,^30^ its applicability within India’s unique social milieu remains less explored.^31^ This study therefore aims to contribute to the validation of the FertiQoL as a reliable and valid tool for assessing QoL in Indian populations.

## Methods

### Research question

The primary research question addressed in this manuscript is: What is the QoL of women experiencing delayed conception in low-to-mid-socioeconomic neighbourhoods in Delhi, India?

### Study design

The above research question was part of a mixed-methods study that explored three questions: (1) What are the characteristics of women encountering delayed conception?, (2) What is the QoL and mental health of women experiencing delayed conception? and (3) What are the experiences and actions taken by women facing delayed conception? The study protocol is available elsewhere.^32^ In this paper, we report findings related to the QoL of married women who experience delay in conception in Delhi, India. Delayed conception was operationalised as the failure to conceive after 18 months of regular, unprotected sexual intercourse, in line with the study’s inclusion criteria. This criterion was selected to ensure the inclusion of all women who experienced prolonged conception challenges while being followed in the Women and Infants Integrated Interventions for Growth Study (WINGS). This mixed-methods study with a cross-sectional design was nested in a larger randomised study known as the WINGS, which is described in a separate protocol.^33^ A cross-sectional design was chosen due to feasibility and resource constraints, as well as the need to capture baseline insights into the QoL of women experiencing delayed conception within the study timeframe. Given the limited research on this topic in India, this design was considered suitable for an initial investigation before exploring longitudinal methods.

### Survey instrument

To assess the QoL of married women who did not conceive after 18 months of unprotected sexual intercourse, quantitative data were collected using the FertiQoL scale.^34^ The FertiQoL questionnaire is a validated instrument developed by an international collaboration of experts, for specifically assessing the QoL in all people experiencing fertility problems.^26^ As opposed to a generic measure of QoL, it is specific to infertility or delayed conception. This scale consists of 36 items comprising two modules, a core module (24 items) and an optional treatment module (12 items). For this study, the treatment module was excluded as the study did not formally assess infertility/delayed conception treatment.

The core module assesses the impact of infertility/delayed conception on four domains, that is, mind-body (six items), relational (six items), social (six items) and emotional (six items). It assesses these impacts on different formats of a five-point Likert scale: (1) from very poor to very good (one item); (2) from very dissatisfied to very satisfied (seven items); (3) from completely to not at all (four items); (4) from always to never (eight items) and (5) from an extreme amount to not at all (six items).

The overall scores and subscale scores for QoL are therefore derived using 24 core items representing mind-body, relational, social and emotional domains. The emotional subscale score assesses the impact of negative emotions (eg, jealousy and resentment, sadness, depression) on QoL. The mind-body subscale score evaluates the impact of fertility problems on physical health (eg, fatigue, pain), cognitions (eg, concentration) and behaviour (eg, disrupted daily activities, delayed life plans). The relational subscale score gauges the impact of fertility problems on the marriage or partnership (eg, sexuality, communication, commitment). The social subscale score measures the extent to which social interactions have been affected by fertility problems (eg, social inclusion, expectations, stigma and support). Two additional items capture an overall evaluation of physical health and satisfaction with QoL. Total scaled scores range from 0 to 100, with higher scores indicating better QoL.^34^ The core module of the scale and its scorings are attached as online supplemental annexure 1.

### Study population

As described in this study’s protocol,^32^ the study population comprised all married women who completed 18 months of unprotected sexual intercourse without getting pregnant while being followed up in a separate randomised study, known as WINGS. WINGS was an individually randomised factorial design trial to assess the impact of a package of community-level interventions delivered pre-conception and post-conception on birth outcomes and infant growth. In WINGS, married women aged 18–30 years, living with their husband with no child or one child, and wanting to have more children, are enrolled and randomised to receive the pre-conception intervention package or routine care, until they are identified to be pregnant or until 18 months’ post-enrolment. Although the study was conducted in Delhi, the sampled population represents socioeconomically diverse urban and peri-urban communities, like those found in other parts of Northern India. Therefore, the findings may be broadly applicable to similar populations within the region, though generalisability should be considered with caution.

### Recruitment and informed consent procedures

All married women who completed 18 months in WINGS without getting pregnant were contacted by field workers, and the purpose of this study was explained to them. As this is a distinct study from WINGS, study participants were contacted at least 14 days after their exit from WINGS.^32^ Married women who were interested in participating were given an appointment during which detailed information about the study was provided by research assistants and informed consent was obtained.

The research assistants, who are nurses by training, checked that the women understood the purpose of the study, its benefits and risks. They provided women with detailed information about the study’s aims and potential benefits before their enrolment. To ensure that women were not under duress to consent, clear statements were provided explaining to the women that their participation was not obligatory but entirely voluntary. Written consent was obtained from all participants, who were reassured that their personal information would be kept confidential throughout the study. Once informed written consent was obtained, face-to-face administration of the survey questionnaire was conducted. In the end, a total of 1530 married women participated.

### Outcomes

The primary outcome of this study is QoL as assessed as a continuous outcome through FertiQoL scores. The details of the scale are provided in online supplemental annexure 1.

### Exposure variables

The exposure variables included social demographic characteristics of the married women and their families (such as age, education, occupation, religion, duration of marriage, wealth index, fertility intentions, substance use, and medical and sexual history). These were selected and adapted from previous surveys and studies related to delayed conception.

### Data analysis

Data were assessed for completeness and cleaned and subsequently analysed using STATA V.16.0 (Stata Corp, Texas, USA). First, sample characteristics were summarised using mean and SD for continuous variables, and proportions for categorical variables. Quintiles were used to categorise variables such as wealth, into least poor, less poor, poor, very poor and poorest. Prevalence estimates (for probable depression and probable anxiety) overall, and by age, education, employment, duration of marriage, wealth quintiles and other demographic variables were derived.

Linear regression was performed to assess the factors associated with QoL. Variables that were found to have a significant association (p<0.2) in univariable analysis were included in the multivariable regression model. The variables with p>0.05 were removed from the multivariable regression model by following the stepwise backward method. The final multivariable regression model consisted of statistically significant variables (p value <0.05) for which the assumptions of the linear regression model were checked. For the regression analysis, we grouped ‘least poor’ and ‘less poor’ quintiles into one category, ‘poor’ as a second category and ‘very poor’ and ‘poorest’ were combined into a third category. This was done to simplify the socioeconomic classification system and facilitate an interpretable analysis.^35^ Confounding was assessed by checking for a change of ≥10% in the beta coefficient when adding or removing covariates. The final adjusted model included key sociodemographic factors, fertility-related variables and experiences of domestic violence and financial stressors, ensuring robust estimates of their independent associations with QoL. Additionally, to explore potential disparities, subgroup analyses were performed by stratifying the sample by socioeconomic status and education level, helping to understand economic constraints and literacy levels’ relationship between delayed conception and QoL perceptions.

Cronbach’s alpha was calculated to assess the overall internal consistency of the FertiQoL scale*,* with values ≥0.7 considered acceptable, 0.6–0.7 considered moderate and values <0.6 indicating low reliability. Additionally, construct validity, a statistic that measured how well the items within the scale collectively reflected the same underlying construct (QoL), was conducted. Confirmatory factor analysis (CFA) was conducted in two stages to assess the factor structure of the FertiQoL scale. First, a first-order CFA evaluated if each item loaded significantly onto its corresponding core domain factor (emotional, mind-body, relational, social). Then, a second-order CFA was built on this by examining how these core domain factors contributed to a higher-order latent construct representing overall FertiQoL. Goodness-of-fit for both models was assessed by using indices like Comparative Fit Index (CFI) and Root Mean Square Error of Approximation (RMSEA), aiming for values indicative of good fit (CFI ≥0.90, RMSEA <0.08). This comprehensive CFA approach ensured a rigorous evaluation of the FertiQoL scale’s psychometric properties in the targeted Indian population.

## Results

Table 1 displays the baseline characteristics of the 1530 married women enrolled in this study. The women had a mean age of 26.8 (SD=3.3) years and an average education duration of 10.2 (SD=4.3) years. Only 6.0% (91/1530) were employed, with the majority being housewives. About 48.8% (746/1530) had at least one living child, whose mean age was 47.1 (SD=29.72) months. Approximately 40% (887/1530) fell into the bottom two quintiles of the wealth index. The average annual income was $2801.2 (SD=$1405.5), with a median of $2400.1 (IQR= $1800.1 to $3360.1). Nearly all households (90.4%; 1384/1530) had bank accounts, while only 11.8% (180/1530) had health insurance. All enrolled households had essential amenities like concrete roofs, toilets, water connections and legal electricity connections.

**Table 1:** Baseline characteristics of the married women included in the study

Characteristic	N=1530
Women’s age (in years), Mean (SD)	26.79 (3.3)
Women with at least one living child	746/1530 (48.8)
Child’s age (in months) (n=746), Mean (SD)	47.05 (29.7)
Household structure	
Proportion living in	
Nuclear family, n/N (%)	661/1530 (43.2)
Extended or joint family, n/N (%)	869/1530 (56.8)
Total number of household members, Mean (SD)	4.83 (2.7)
Women’s years of schooling (years), Mean (SD)	10.16 (4.3)
Never been to school, n/N (%)	80/1530 (5.2)
Women’s current occupation	
Employed,* n/N (%)	91/1530 (6.0)
Housewife, n/N (%)	1439/1530 (94.1)
Husband’s age in years, Mean (SD)	28.62 (3.9)
Husband’s years of schooling, Mean (SD)	10.99 (3.8)
Never been to school, n/N (%)	43/1530 (2.8)
Husband’s current occupation	
Government service, n/N (%)	14/1530 (0.9)
Private sector job, n/N (%)	1094/1530 (71.5)
Daily wager/labourer, n/N (%)	68/1530 (4.4)
Self-employed, n/N (%)	290/1530 (20.0)
Does not work, n/N (%)	64/1530 (4.2)
*Household information*	
Religion of the head of the household, n/N (%)	
Hindu	1268/1530 (82.9)
Muslim	238/1530 (15.6)
Others	24/1530 (1.5)
A member of the household owns this house or any other house, n/N (%)	1171/1530 (76.5)
A member of the household owns this house or any other house, n/N (%)	
Male	965/1171 (82.4)
Female	199/1171 (17.0)
Joint	7/1171 (0.6)
A member of the household having a bank account or a post office account, n/N (%)	1384/1530 (90.5)
Households having a below-poverty-line card, n/N (%)	73/1530 (4.8)
Households having health schemes or health insurance, n/N (%)	180/1530 (11.8)
Socioeconomic details	
Annual family income (in US$), Mean (SD)	2801.21 (1405.5)
Wealth quintiles, n/N (%)	
Poorest	306/1530 (20.0)
Very poor	306/1530 (20.0)
Poor	309/1530 (20.2)
Less poor	303/1530 (19.8)
Least poor	306/1530 (20.0)

* Government service, private job, daily wage/labourer, self-employed.

† Other religions: Christian, Jain and Sikh.

Table 2 provides an overview of the general history and fertility intentions of the female participants in the study. Almost all women in the study were married, and the average age at the birth of their first child was 22.2 (SD=2.7) years, and a small percentage had adopted, fostered or had stepchildren (3.07%). Women reported trying to conceive for an average of 3.2 (SD=2.1) years. Over half of the women reported having unprotected sexual intercourse more than two times a week.

**Table 2:** Reproductive history and fertility intentions of the women

Variables	N=1530
Women who never had a child, n/N (%)	755/1530 (49.4)
Women who have any adopted, fostered or stepchildren, n/N (%)	47/1530 (3.1)
Maternal age in years at first birth, Mean (SD)	22.15 (2.7)
Years woman has been married to her current husband, Mean (SD)	6.42 (3.2)
Partner fathered a child (from another woman)	626/1530 (40.9)
Intended pregnancy before enrolment in the primary trial*	1224/1530 (80.0)
Duration of trying to conceive (in years), Mean (SD)	3.21 (2.1)
Unprotected sexual intercourse	
Once every month	27/1530 (1.8)
Two times per month	100/1530 (6.5)
Once a week	199/1530 (13.0)
Two times per week	433/1530 (28.3)
More than two times per week	771/1530 (50.4)
Reports delay in pregnancy (as per woman)	1374/1530 (89.8)
Reports delay in pregnancy (as per husband)	763/1530 (49.9)
Experienced from partner	
Physical abuse	62/1530 (4.1)
Emotional abuse	262/1530 (17.1)
Verbal abuse	131/1530 (8.6)
Denial of financial support	34/1530 (2.2)
Divorce	30/1530 (2.0)
Abandonment	31/1530 (2.0)
Experienced from anyone else in the family for taking too long to become pregnant	
Physical abuse	47/1530 (3.1)
Emotional abuse	748/1530 (48.9)
Verbal abuse	279/1530 (18.2)
Denial of financial support	51/1530 (3.3)
Divorce	41/1530 (2.7)
Abandonment	51/1530 (3.3)

* The participants of this study were earlier a part of trial names as WINGS.

WINGS, Women and Infants Integrated Interventions for Growth Study.

Additionally, 89.8% (1374/1530) of women perceived a delay in pregnancy, while only around half (49.9%, 763/1530) of the husbands, as reported by the women, also perceived a delay. This may reflect varying expectations or pressures to conceive, suggesting that women might expect conception within a shorter period compared with men. Among the married women surveyed, 4.1% (62/1530) reported experiencing physical abuse from their partners, while emotional and verbal abuse was more common, reported by 17.1% (262/1530) and 8.6% (131/1530) of women, respectively. Additionally, 2.2% (34/1530) reported being denied financial support by their partners, and 2.0% experienced abandonment (31/1530). Abuse from other family members was even more prevalent, particularly emotional abuse, reported by nearly half of the women (48.9%, 748/1530). Verbal abuse was experienced by 18.2% (279/1530), and physical abuse by 3.1% (47/1530). Financial neglect, divorce and abandonment by family members were reported by 3.3% (51/1530), 2.7% (41/1530) and 3.3% (51/1530) of women, respectively.

Table 3 offers insights into the QoL for women experiencing delayed conception, as measured by the scores of FertiQoL scale (ranges from 0 to 100), with higher scores indicating better QoL. The average scaled FertiQoL score of 31.7 (out of 100) suggests a moderately lower level of QoL compared with the general reference population. This is further confirmed by the median score of 32.3. In the emotional domain, the mean score of 29.03 and median of 29.2 reveal a moderate level of emotional well-being, with a wider deviation (SD=11.1) indicating individual variation. In the mind-body domain, encompassing physical health and body image shows a mean score of 25.4 and median of 25, again suggesting moderate well-being with some variation. The relational domain scores are higher, with a mean of 50.7 and median of 58.3. However, the presence of some variation again points to individual experiences. In the social domain, the mean score of 21.7 and median of 20.8 suggest a moderately lower level of satisfaction with social interaction and activities.

**Table 3:** Total raw scores of FertiQoL and its subscales for the women having delayed conception

Quality of life domain	Scaled scores
General physical health score	
Mean (SD)	57.5 (22.0)
Median (IQR)	50 (50–75)
General life satisfaction	
Mean (SD)	44.58 (24.11)
Median (IQR)	50 (25–50)
FertiQoL scores	
Mean (SD)	31.7 (7.1)
Median (IQR)	32.3 (28.1–36.5)
*SUBSCALES scores of FertiQoL*	
Emotional domain	
Mean (SD)	29.0 (11.1)
Median (IQR)	29.2 (20.8–37.5)
Mind-body domain	
Mean (SD)	25.4 (8.30)
Median (IQR)	25 (20.8–33.3
Relational domain	
Mean (SD)	50.7 (13.8)
Median (IQR)	58.3 (45.8–58.3)
Social domain	
Mean (SD)	21.7 (10.1)
Median (IQR)	20.8 (12.5–29.2)

FertiQoL, Fertility Quality of Life.

The internal consistency among the items of the scale was found to be 0.64 (Cronbach alpha score) and when we removed eight items (Q7, 8, 12, 5, 10, 13, 14 and 3) the coefficient raised to 0.68 (online supplemental eTable 1). The removal of these items from the scale was likely considered to improve its internal consistency, as measured by Cronbach’s alpha. However, the difference between Cronbach’s alpha of 0.64 and 0.68 was relatively small and not considered substantial. Removing these items could compromise the comprehensiveness of the scale, as they represent important aspects of the construct being measured. Furthermore, since this was the first time the validity of the FertiQoL scale was assessed in India, we prioritised the theoretical framework underlying the scale, which emphasises that all items contribute to capturing the multifaceted impact of infertility/delayed conception. Removing items could potentially disrupt the balance across its subdomains. Additionally, the CFA demonstrated good fit indices for the hierarchical factor model under examination (CFI=0.90; Standardised root means square residual=0.05). The first-order factor loadings for each Core FertiQoL domain are presented in online supplemental eTable 2, while online supplemental eFigure 1 illustrates the second-order factor loadings between the core FertiQoL domains. Notably, both the first and second-order factor loadings were statistically significant (p<0.001), with the majority hovering around or exceeding 0.30.

Online supplemental eGraphs 1–4 display the distribution of responses in the emotional, mind-body, social and relational domains of the FertiQoL questionnaire, indicating the percentage of participants for each level of emotional experience. The data reveal a wide range of experiences (green bar showing the percentages of responses for different items of FertiQoL) among women facing delayed conception.

Table 4 shows the adjusted linear regression analysis which explores the relationship between various factors and FertiQoL scores, which represent the QoL of participants. The adjusted QoL coefficients, along with their 95% CIs and p values, provide an estimate of the impact of each independent variable on FertiQol scores.

**Table 4:** Findings from univariable and multivariable linear regression for factors determining quality of life (QoL) among women with delayed conception

Variables	Adjusted FertiQoL-coefficient (95% CI) (p value)
*Partnership and children’scircumstances*	
Husbands had fathered any other children (from another woman/outside marriage)	
Yes	−1.1** (−1.70, –0.38) (p=0.00)
No	Reference
*Fertility intentions*	
Women trying to get pregnant over 18 months (ie, prior to joining the primary trial).	
Yes	−0.3** (−0.5, –0.1) (p=0.00)
No	Reference
Perception of the woman that conception is taking longer	
Yes	−1.2* (−2.3, –0.2) (p=0.02)
No	Reference
Women felt isolated	
Yes	−1.6** (−2.3, –0.9) (p=0.00)
No	Reference
Women are emotionally abused by partners/husband	
Yes	−3.5** (−4.5, –2.4) (p=0.00)
No	Reference
Women verbally abused by their partner/husband	
Yes	−3.2** (−4.7, –1.7) (p=0.00)
No	Reference
Women abandoned by partner/husband	
Yes	−4.8** (−7.3, –2.4) (p=0.00)
No	Reference
Women are physically abused by their family members	
Yes	−3.6** (−5.5, –1.6) (p=0.00)
No	Reference
Women verbally abused by family members	
Yes	−1.2* (−2.1, –0.2) (p=0.02)
No	Reference
Women denied financial support by their family members	
Yes	−3.2* (−5.2, –1.2) (p=0.004)
No	Reference
*Sexual history of the women*	
Abnormal vaginal discharge	
Yes	−0.9* (−1.7, –0.2) (p=0.01)
No	Reference

** p value less than/equal to= 0.00; * p value > 0.00 but <=0.05.

FertiQoL, Fertility Quality of Life.

Results show that several factors were significantly associated with QoL. Women who had been trying to become pregnant for a long duration had a negative impact on their QoL, as did women whose husbands had fathered other (non-biological) children, (QoL coefficient=−0.3, 95% CI: −0.5 to −0.1, p=0.00) and (QoL coefficient=−1.0, 95% CI: −1.7 to −0.4, p=0.00), respectively. Women who perceived that she was taking a longer time to become pregnant had 1.2 (95% CI: −2.3 to −0.2, p=0.02) lower QoL scores compared with women who did not perceive that they were taking a longer time. Furthermore, domestic violence from husbands or family members had a significant negative impact on the QoL life scores. Women who felt isolated (QoL coefficient=−1.6, 95% CI: −2.3 to −0.9, p=0.00), experienced emotional abuse by their husbands (QoL coefficient=−3.5, 95% CI: −4.5 to −2.4, p=0.00) and were verbally abused by their partner/husband (QoL coefficient=−3.2, 95% CI: −4.7 to −1.7, p=0.00), abandoned by their husbands (QoL coefficient=−4.8, 95% CI: −7.3 to −2.4, p<0.0001) had significantly lower total FertiQoL scores. In addition, abuse by family members also contributed to significantly lower scores. Women who were physically abused (QoL coefficient=−3.6, 95% CI: −5.5 to −1.6, p=0.00), verbally abused (QoL coefficient=−1.2, 95% CI: −2.1 to −0.2, p=0.02) and denied financial support (QoL coefficient=−3.2, 95% CI: −5.2 to −1.2, p=0.004) by family members had lower QoL. Furthermore, the women experiencing abnormal vaginal discharge had significantly lower FertiQol scores (B=−0.9, 95% CI: −1.7 to −0.2, p=0.01) compared with women without such symptoms. The findings for unadjusted and adjusted linear regression analysis can be found in online supplemental eTable 3.

## Discussion

This cross-sectional study reports the QoL of women with delayed conception in the low-to-mid-socioeconomic neighbourhoods of North India and its correlates. The average FertiQoL score was 31.71, with domain scores ranging from 21.70 (social) to 50.68 (relational). The scale showed moderate internal consistency (Cronbach’s alpha=0.64), and CFA demonstrated good fit indices, with most factor loadings exceeding 0.30. Lower scores on the FertiQoL scale were associated with longer duration of delayed conception, husband fathering non-biological children, experience of domestic violence from husband or family in the form of emotional, verbal and physical abuse and isolation, lack of financial support and physical health issues like abnormal vaginal discharge.

Our findings underscore the role of stigma and societal expectations in exacerbating the negative impact of delayed conception on women’s QoL. In many cultures, particularly in South Asia, delayed conception is often viewed as a personal failure of women, subjecting them to blame, exclusion and psychological distress.^8 9^ Women who struggle to conceive may face intense familial and societal pressure, leading to stress, anxiety and in some cases, domestic violence.^36 37^ This aligns with studies from other LMICs where infertility or delayed conception-related stigma affects mental health, self-worth and marital stability.^37^ Additionally, social exclusion, particularly in patrilocal households, further isolates women from familial support, increasing their emotional burden.^18^ Addressing these deeply rooted social norms through community education and counselling interventions could help mitigate the stigma associated with delayed conception.

Our findings are broadly like those from other studies conducted in Zanzibar, Kazakhstan and Turkey, which also showed moderately lower QoL with lower emotional and mind-body domain scores, compared with the remaining two domains.^36 38 39^ Notably, the emotional and mind-body domains were the most affected, scoring the lowest at 29.0 (SD=11.1) and 25.4 (SD=8.3), respectively. These findings indicate that delayed conception-induced distress, negative emotions and perceived physical health issues are major contributors to impaired QoL. Conversely, the relational domain exhibited the highest scores (mean=50.7, SD=13.8), suggesting that marital relationships were less impacted compared with other QoL dimensions. However, variability in scores suggests differences in how couples navigate fertility-related stress, with some maintaining relational stability while others experience strain. The social domain scored the lowest (mean=21.7, SD=10.1), highlighting the profound impact of stigma and social exclusion on women’s well-being. A recent Indian study conducted in the outpatient department echoed a similar trend, with the least impact on the relational domain.^40^ However, cultural differences may shape how delayed conception affects QoL. For example, studies in high-income countries, such as the USA and the Netherlands, report that while infertility or delayed conception affects mental well-being, the presence of social support networks and equitable gender norms reduce its impact on marital relationships and personal identity.^8 27^ In contrast, in settings where childbearing is closely tied to a woman’s societal status and economic security, delayed conception has a more profound effect on social interactions and emotional well-being.^19 40 41^ These crosscultural differences highlight the need for context-specific interventions that address both the psychological and societal dimensions of delayed conception.

Our findings are also consistent with other studies reporting that there is a longer duration that women are trying to conceive and failing negatively affects QoL.^38^,^40^ Additionally, our findings are also consistent with other studies demonstrating the negative effect of violence on QoL among populations of infertile^41 42^ as well as general population women.^43^

Our findings align with previous studies that highlight the association between longer durations of attempting to conceive and reduced QoL.^44^ Similarly, our results are consistent with research demonstrating the adverse impact of violence on QoL, both among infertile populations and women in the general population.^37 40 41^ Additionally, economic hardship was significantly associated with lower QoL scores, which is found in other studies too.^10^ Women from households in the poorest wealth quintile had lower QoL scores compared with those in the least poor quintile. Similarly, women who lacked financial support from their families had significantly lower FertiQoL scores (β=−3.2, 95% CI: −5.2 to −1.2, p=0.004). These findings highlight how financial insecurity exacerbates delayed conception-related distress, likely due to the high costs of medical consultations and treatments. Stigma also played a major role in shaping QoL perceptions, also highlighted in other studies.^45^ Women who felt socially isolated had significantly lower QoL scores (β=−1.6, 95% CI: −2.3 to −0.9, p<0.001), reinforcing the social consequences of delayed conception. Moreover, women who perceived a delay in conception had 1.2 (95% CI: −2.3 to −0.2, p=0.02) lower QoL scores than those who did not perceive a delay, emphasising the psychological burden of prolonged delayed conception.

The FertiQoL scale has Cronbach’s alpha of 0.64 and the recent publications by van Griethuijsen and Taber consider alpha values within the range of 0.70–0.60 could be considered acceptable.^46 47^ After we isolated eight items (Q7, 8, 12, 5, 10, 13, 14 and 3) in a sensitivity analysis, the coefficient raised to 0.68; nevertheless we did not delete any item from the scale as alpha values of 0.70–0.60 are acceptable or adequate as per available literature.^46 47^ Given that the FertiQoL scale is in its early stages of research in India, this alpha level can be tolerated and indicates a reasonable correlation among items measuring QoL in the context of delayed conception in India. However, it is worth noting that a few values calculated for Cronbach’s alpha for individual domains of FertiQoL are below the acceptable thresholds of 0.7 or 0.6. We posit that this may be due to the limited number of items contributing to the factors. The future focus can now shift towards gathering qualitative feedback and conducting further testing to enhance the scale’s reliability, particularly the Hindi version of the scale. Our study showed statistically significant (p<0.001) factor loadings for both first and second-order factors, which supports the model’s efficacy in capturing specific QoL facets (emotional, mind-body, social and relational) and the broader domain of QoL among those experiencing delayed conception. This assertion is also supported by the strong associations (factor loadings >0.30) identified in our study as it aligns with the suggested cut-off value for accepting a factor loading.^48^ Good CFA results, with a CFI of 0.90 and SRMR of 0.05, affirm the proposed hierarchical factor model’s alignment with observed data, enhancing the construct validity and generalisability of findings regarding the complex interrelationships within QoL domains and facets in the context of delayed conception.

### Implications for health and social services, and future research

Our findings have implications related to supportive interventions for women who are encountering delayed conception and specifically indicate the need for culturally sensitive psychosocial interventions and support systems for this group of women. These interventions should not only focus on emotional and psychological well-being but also address social and economic stressors faced by individuals dealing with delayed conception in India. This should include interventions that reduce couple and family violence. Evidence shows that violence against women who are failing to conceive contrary to family and spousal expectations is common, particularly in low and middle-income countries.^37^ Recent studies have shown that integrating couple-based interventions, such as joint counselling and gender-sensitive education, can help reduce domestic violence and improve marital support.^49 50^ In this context, implementing couple-based counselling interventions may contribute to resolving or reducing domestic violence and mistreatment, which is often driven by a belief that women are solely responsible for achieving or failing to achieve pregnancy in our study community^24^ and other settings.^51^ Studies show that support from spouses, family and wider social networks is essential in mitigating negative experiences among such women.^49 52^

Additionally, financial strain emerged as a key factor impacting QoL, reinforcing the importance of affordable and accessible infertility/delayed conception services. The Indian government has made strides in regulating and expanding fertility services,^53^ but awareness and accessibility remain challenges. A recent global systematic review showed that financial constraints significantly limit access to fertility treatments, with lower-income women disproportionately affected.^10^ Also, in many settings, help-seeking among couples who are struggling to conceive is very low, partly because of stigma and lack of awareness.^12^ Efforts to increase public awareness, reduce stigma and integrate delayed conception care into existing reproductive health services could improve help-seeking behaviour and QoL outcomes.

In terms of future research, our study has two key implications. First, the Cronbach’s alpha for some individual domains falls below the ideal threshold, suggesting the need for further research to refine the Hindi version of the scale and improve its reliability. Second, future studies should explore QoL among men in couples experiencing delayed conception. Men are often overlooked in infertility/delayed conception research, despite evidence that male infertility/delayed conception also carries significant psychological and social burdens.^19 54^ Understanding the experiences of both partners would provide a more holistic view of the challenges faced by couples and inform more inclusive intervention strategies.

### Limitations and strengths

Our study’s cross-sectional design does not establish causal relationships between variables and QoL, for which other study designs would be better suited. However, by identifying factors associated with QoL, our study provides useful insights into potential interventions that could mitigate the negative effects of delayed conception, including those that can strengthen individual and collective coping and resilience mechanisms. Future longitudinal studies could provide a clearer picture of how QoL evolves over time among women experiencing delayed conception. Our study focuses solely on women, potentially limiting insights from male partners and as mentioned above, future research should aim to generate insights from men who are facing delays in achieving pregnancy. Additionally, our reliance on self-reported data by women may introduce recall and social desirability biases, as participants may underreport distress or violence due to societal stigma. To minimise recall and social desirability bias, we assured participants of complete confidentiality, ensuring that their responses would not be shared with family members, community members, or healthcare providers. Additionally, we used non-judgmental phrasing to normalise experiences and trained female interviewers to ask sensitive questions in a standardised and empathetic manner, fostering trust and encouraging honest responses. Nevertheless, our large sample of over 1500 women recruited from community settings enhances the generalisability of our findings and boosts confidence in the statistical results. Moreover, this is the first study to assess the reliability and validity of FertiQoL in the Indian context.

## Conclusion

This research enhances our understanding of the intricate connection between delayed conception and QoL. The established model and its findings provide critical insights that can inform culturally sensitive psychosocial interventions to address the emotional, social and economic challenges faced by women experiencing delayed conception. Healthcare providers could integrate these findings into routine care by incorporating mental health screenings, providing targeted counselling and facilitating peer support groups to help women navigate delayed conception-related issues. Additionally, couple-based interventions addressing domestic violence offer promising avenues for improving marital dynamics and emotional well-being.

From a policy perspective, government initiatives could expand financial support programmes tailored for low-to-mid-socioeconomic populations dealing with infertility/delayed conception, ensuring equitable access to fertility services and reducing economic barriers to care. Further research should explore scalable models for integrating fertility-related mental health interventions into primary healthcare settings. This study’s large sample size and strong psychometric evaluation reinforce the generalisability and validity of its conclusions, contributing significantly to the understanding of delayed conception’s complex impact on the QoL of married women in India and guiding future intervention strategies.

## Supplementary material

10.1136/bmjph-2024-001740 online supplemental file 1

10.1136/bmjph-2024-001740 online supplemental file 2

## Data Availability

Data are available upon reasonable request.
