# Supplementary material for: Quality of life and its determinants in women with delayed conception in low-mid socioeconomic neighbourhoods of Northern India: a cross-sectional study
Source: BMJ Public Health. 2025 Apr 20;3(1):e001740. doi: 10.1136/bmjph-2024-001740 (PMC12010299; doi:10.1136/bmjph-2024-001740)
Supplement: online supplemental file 1 [file bmjph-3-1-s001.pdf]

See full details www.fertiqol.org

FertiQoL scoring

Response categories: FertiQoL consists of 36 items scored according to 5 response categories. The response scale has a range of 0 to 4. Higher scores mean higher quality of life. Categories and scales are:

Response category	Scale
Evaluation	Very poor (0), Poor (1), Nor good nor poor (2), Good (3), Very good (4)
Satisfaction	Very dissatisfied (0), Dissatisfied (1), Neither satisfied nor dissatisfied (2), Satisfied (3), Very satisfied (4)
Frequency	Always (0), Very often (1), Quite often (2), Seldom (3), Never (4)
Intensity	An extreme amount (0), Very much (1), A moderate amount (2), A little (3), Not at all (4)
Capacity	Completely (0), A great deal (1), Moderately (2), Not much (3), Not at all (4)

Subscale and total scales:

FertiQoL yields six subscale and three total scores with a range of 0 to 100.
Two additional items (marked A and B on the FertiQoL questionnaire) capture an overall evaluation of physical health and satisfaction with quality of life and are not used in FertiQoL scoring.

The **Core FertiQoL** is the average fertility quality of life across all domains. The subscales for the Core FertiQoL are **Emotional, Mind-Body, Relational and Social** subscales. The **Emotional** subscale score shows the impact negative emotions (e.g., jealousy & resentment, sadness, depression) have on quality of life. The **Mind-Body** subscale score shows the impact infertility has had on physical health (e.g., fatigue, pain) cognition (e.g., concentration) and behaviour (e.g., disrupted daily activities, delayed life plans). The **Relational** subscale score shows the impact fertility problems have had on the components (e.g., sexuality, communication, commitment) of relationship or partnership. The **Social** subscale score shows the extent to which social interactions have been affected by fertility problems (e.g., social inclusion, expectations, stigma, support).

The **Treatment FertiQoL** is the average quality of life across treatment domains. The treatment subscales are **Treatment Environment and Treatment Tolerability**. The **Treatment Environment** subscale score shows the extent to which the accessibility and quality of your treatment has impacted your quality of life. The **Treatment Tolerability** subscale score shows the extent to which you have experienced mental and physical symptoms as a result of your fertility treatment and the impact this has had on daily life. The **Total FertiQoL** score is the average quality of life for all core and treatment domains.

	***Core* FertiQoL**				***Treatment* FertiQoL**	
	Emotional	*Mind/Body*	*Relational*	*Social*	*Environment*	*Tolerability*
Item	Q4R	Q1	Q6	Q5	T2R	T1
	Q7	Q2	Q11R	Q10	T5R	T3
	Q8	Q3	Q15R	Q13	T7	T4
	Q9	Q12	Q19	Q14R	T8	T6
	Q16	Q18	Q20	Q17	T9	
	Q23	Q24	Q21R	Q22	T10	

Note. Item number refers to item number on the FertiQoL questionnaire. Items marked 'Q' are Core FertiQoL items and those marked T are Treatment FertiQoL items. Items marked with an R need to be reversed before summing. For these items use the reverse of the response scale (4 to 0, instead of 0 to 4) so that higher scores reflect higher quality of life.

Subscale and total scoring:

Scoring consists of three steps.

1) Reverse items.

2) Calculate raw scores by summing all items that belong to the subscale or total scale. For the Core FertiQoL add all ‘Q’ items (24 items). For the Treatment FertiQoL add all the ‘T’ items (10 items). For the Total FertiQoL add all Core and Treatment items (34 items).

3) To compute scaled scores for the subscale and total scales, multiply the relevant raw score by 25/k, where k is the number of items in the subscale. The scaled scores range is 0 to 100.

Scoring example

Scoring example

Suppose Mary had given the following scores to the emotional items.

Q4R = 0 Q7 = 3 Q8 = 2 Q9 = 2 Q16 = 3 Q23 = 2

To calculate the Emotional subscale score (scaled):

Step 1: Reverse item Q4R. *** Q4R reverse is 4.
Step 2 = Add all subscale items: *(4 + 3 + 2 + 2 + 3 + 2) = 16*
Step 3 = Multiply raw score by 25/k, where k is the number of items:

16*(25/6)

Mary's scaled Emotional subscale score = 66.7
