# Supplementary material for: Quality of life and its determinants in women with delayed conception in low-mid socioeconomic neighbourhoods of Northern India: a cross-sectional study
Source: BMJ Public Health. 2025 Apr 20;3(1):e001740. doi: 10.1136/bmjph-2024-001740 (PMC12010299; doi:10.1136/bmjph-2024-001740)
Supplement: online supplemental file 2 [file bmjph-3-1-s002.docx]

**Quality of Life and its Determinants in Women with Delayed Conception in Low-Mid Socioeconomic Neighbourhoods of Northern India**

Barsha Gadapani Pathak,^1,^ ^3^ Gitau Mburu,^2^ Ndema Habib,^2^ Rita Kabra,^2^ James Kiarie,^2^ Ranadip Chowdhury,^1^ Neeta Dhabhai,^1^ Sarmila Mazumder^1^*

1 Society for Applied Studies, New Delhi, India

2 UNDP-UNFPA-UNICEF-WHO-World Bank Special Program of Research, Development and Research Training in Human Reproduction (HRP) Department of Sexual and Reproductive Health and Research, World Health Organization, Geneva, Switzerland.

3 Centre for International Health, Faculty of Medicine, University of Bergen, Norway

**Corresponding author:** Sarmila Mazumder, Society for Applied Studies, New Delhi, India Email: [sarmila.mazumder@sas.org.in](mailto:sarmila.mazumder@sas.org.in)

**Supplementary Table, figures, and Graphs**

**Supplementary Table 1:** The Reliability coefficients (Cronbach’s coefficient) for the total and subscales of the FertiQoL scale.

**Supplementary Table 2:** First-order factor loadings on the FertiQoL scale.

**Supplementary Figure 1**: Fertility quality of life second-order factors.

**Supplementary Graph 1:** The scorings of the emotional domain of quality of life assessed by the FertiQol scale.

**Supplementary Graph 2:** The scorings of the social domain of quality of life assessed by the FertiQol scale.

**Supplementary Graph 3:** The scorings of the mind-body domain of quality of life assessed by the FertiQol scale.

**Supplementary Graph 4:** The scorings of the relational domain of quality of life assessed by the FertiQol scale.

**Supplementary Table 1: The Reliability coefficients (Cronbach’s coefficient) for total and subscales FertiQoL**

| **Subscales of FertiQoL** | Number of items | Cronbach coefficient |
| --- | --- | --- |
| Emotional | 6 | 0.33 |
| Mind/Body | 6 | 0.39 |
| Relational | 6 | 0.74 |
| Social | 6 | 0.12 |
| **Total FertiQoL** | 24 | 0.64 |
| Removing (8 items: Q7, Q8, Q12, Q5, Q10, Q13, Q14, Q3) | 16 | 0.68 |

**Supplementary Table 2:** First-order factor loadings on FertiQoL.

| **Item no**. | **FertiQoL core subscales** | **First order factor loadings** |
| --- | --- | --- |
|  | Emotional |  |
| Q9 | Fluctuate hope/despair | 0.02 |
| Q16 | Sad or depressed mood | 0.98 |
| Q8 | Grief | 0.40 |
| Q23 | Angry | 0.30 |
| Q7 | Jealousy and resentment | 0.13 |
| Q4 | Unable to cope | 0.10 |
|  | Mind Body |  |
| Q3 | Feel worn out | 0.66 |
| Q18 | Fatigue | 0.60 |
| Q2 | Life on hold | 0.24 |
| Q24 | Pain/discomfort | 0.28 |
| Q12 | Disrupt activities | 0.05 |
| Q1 | Impact concentration | 0.03 |
|  | Relational domain |  |
| Q19 | Impacts relationship negatively | 0.20 |
| Q20 | Difficult to talk | 0.14 |
| Q21 | Content relationship | 0.84 |
| Q6 | Satisfied with sexual relationship | 0.38 |
| Q11 | Affectionate | 0.79 |
| Q15 | Strengthen commitment | 0.84 |
|  | Social |  |
| Q13 | Feels inferior | 0.36 |
| Q17 | Social pressure | 0.20 |
| Q10 | Feels isolated | 0.30 |
| Q5 | Friends support | 0.02 |
| Q14 | Understanding of family | 0.05 |

**Supplementary Figure 1: Fertility quality of life second-order factors.**


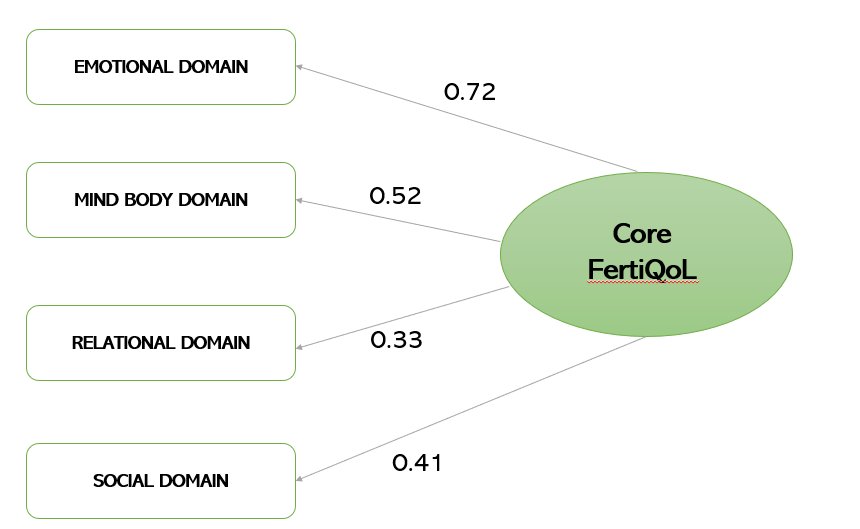
*Numbers next to the arrows represent standardized factor loadings and correlations between factors.*

**Supplementary Graph 1:** The scorings of the emotional domain of quality of life assessed by FertiQol scale.


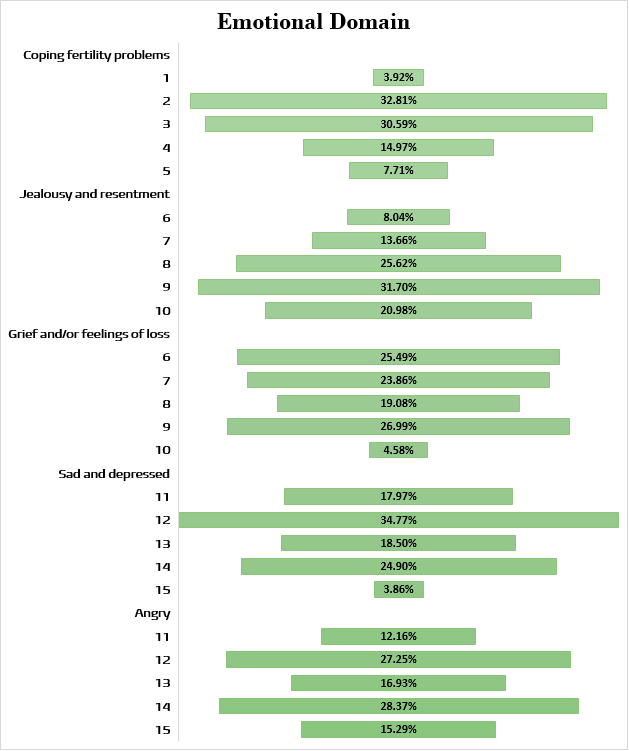


| **EMOTIONAL DOMAIN’s Likert scoring** | | | | | | | | | | | | | | |
| --- | --- | --- | --- | --- | --- | --- | --- | --- | --- | --- | --- | --- | --- | --- |
| **Completely** | **A great deal** | **Moderately** | **Not much** | **Not at all** | **Always** | **Very often** | **Quite often** | **Seldom** | **Never** | **An extreme amount** | **Very much** | **A moderate amount** | **Little** | **Not at all** |
| 1 | 2 | 3 | 4 | 5 | 6 | 7 | 8 | 9 | 10 | 11 | 12 | 13 | 14 | 15 |

**Supplementary Graph 2:** The scorings of the social domain of quality of life assessed by the FertiQol scale.

| **SOCIAL DOMAIN’s Likert scoring** | | | | | | | | | | | | | | |
| --- | --- | --- | --- | --- | --- | --- | --- | --- | --- | --- | --- | --- | --- | --- |
| **Very dissatisfied** | **Dissatisfied** | **Neither Satisfied**  **Nor Dissatisfied** | **Satisfied** | **Very**  **Satisfied** | **Always** | **Very often** | **Quite often** | **Seldom** | **Never** | **An extreme amount** | **Very much** | **A moderate amount** | **Little** | **Not at all** |
| 1 | 2 | 3 | 4 | 5 | 6 | 7 | 8 | 9 | 10 | 11 | 12 | 13 | 14 | 15 |

**Supplementary Graph 3:** The scorings of the mind-body domain of quality of life assessed by the FertiQol scale.

| **Mind-body domain’s Likert scores** | | | | | | | | | | | | | | |
| --- | --- | --- | --- | --- | --- | --- | --- | --- | --- | --- | --- | --- | --- | --- |
| **Completely** | **A great deal** | **Moderately** | **Not much** | **Not at all** | **Always** | **Very often** | **Quite often** | **Seldom** | **Never** | **An extreme amount** | **Very much** | **A moderate amount** | **Little** | **Not at all** |
| 1 | 2 | 3 | 4 | 5 | 6 | 7 | 8 | 9 | 10 | 11 | 12 | 13 | 14 | 15 |

**Supplementary Graph 4:** The scorings of the relational domain of quality of life assessed by the FertiQol scale.

| **RELATIONAL DOMAIN’s Likert scores** | | | | | | | | | | | | | | |
| --- | --- | --- | --- | --- | --- | --- | --- | --- | --- | --- | --- | --- | --- | --- |
| **Very dissatisfied** | **Dissatisfied** | **Neither Satisfied**  **Nor Dissatisfied** | **Satisfied** | **Very**  **Satisfied** | **Always** | **Very often** | **Quite often** | **Seldom** | **Never** | **An extreme amount** | **Very much** | **A moderate amount** | **Little** | **Not at all** |
| 1 | 2 | 3 | 4 | 5 | 6 | 7 | 8 | 9 | 10 | 11 | 12 | 13 | 14 | 15 |

Table 3: Findings from univariable and multivariable linear regression for factors determining quality of life among women with delayed conception

| **Variables** | **FertiQol scores** | |
| --- | --- | --- |
|  | **Unadjusted B-coefficient (95% CI), (p-value)** | **Adjusted B-coefficient (95% CI), (p-value)** |
| **PARTNERSHIP AND CHILDREN’S CIRCUMSTANCES** | | |
| Age of the women | -0.0(-0.1,0.1) (p=0.90) |  |
|  |  |  |
| Women who had at least one [living] child  Yes No | 1.4** (0.7,0.2) (p=0.00)  Reference |  |
| Total number of children that women intend to have in their  lifetime. | 0.6(-0.3, 1.6) (p=0.20) |  |
| Women age at birth of 1st child | -0.2**(-0.0, -0.1) (p=0.00) |  |
| Women have adopted, fostered, or stepchildren. |  |  |
| Yes No | -1.2 (-3.3, 0.9) (p=0.30)  Reference |  |
| Husband’s age | -0.1* (-0.2, -0.1) (p=0.00) |  |
| Duration of marriage (in years) | -0.1*(-0.2, 0.0) (p=0.05) |  |
| Husbands had fathered any other children.(from another woman/outside marriage) Yes  No | 1.0*(0.3,1.70) (p=0.01)  Reference | -1.1**(-1.70, -0.38) (p=0.00) |
| **FERTILITY INTENTIONS** | | |
| Women trying to get pregnant over 18 months (i.e., prior to joining the primary trial).  Yes No | -1.9** (-2.8, -1.1) (p=0.00)  Reference | -0.3** (-0.5, -0.1) (p=0.00)  Reference |
| Total duration trying to conceive | -0.6* (0.8, -0.5) (p=0.00) |  |
| Throughout the WINGS trial, there was a continuous effort to achieve pregnancy.  Yes  No | -1.4* (-2.7, -0.2)  Reference |  |

| Perception of the woman that conception is taking longer. Yes  No | -2.8** (-4.0, -1.6) (p=0.00)  Reference | -1.2*(-2.3, -0.2) (p=0.02)  Reference |
| --- | --- | --- |
